# COVID-19 preparedness and response in the Pitcairn Islands: keeping one of the world’s smallest and most isolated populations safe in a pandemic

**DOI:** 10.5365/wpsar.2024.15.2.1068

**Published:** 2024-06-27

**Authors:** Darralyn Griffiths, Kevin Walters, Sean T Casey

**Affiliations:** a Pitcairn Islands Health Centre, Pitcairn Islands.; b World Health Organization Regional Office for the Western Pacific, Manila, Philippines.; c School of Population Health, University of New South Wales, Sydney, New South Wales, Australia.

## Abstract

**Problem:**

While the COVID-19 pandemic threatened the entire world, the extremely remote Pitcairn Islands faced unique vulnerabilities. With only a physician and a nurse to care for an ageing population of fewer than 40 residents, and with very limited referral pathways, Pitcairn encountered distinct challenges in preparing for and responding to the COVID-19 pandemic.

**Context:**

The Pitcairn Islands is an overseas territory of United Kingdom of Great Britain and Northern Ireland consisting of four islands in the South Pacific: Pitcairn, Henderson, Ducie and Oeno. Pitcairn is the only inhabited island with a local resident population of approximately 31 people, around half of whom were over 60 years old in 2023. The islands are only accessible by sea and are located more than 2000 km from the nearest referral hospital in French Polynesia.

**Actions:**

Pitcairn’s Island Council took aggressive action to delay the importation of SARS-CoV-2, vaccinate its small population and prepare for the potential arrival of the virus.

**Outcomes:**

As of May 2024, Pitcairn was one of the only jurisdictions in the world not to have had a single COVID-19 hospitalization or death. Nevertheless, the pandemic presented the islands’ population with many economic, social and health challenges.

**Discussion:**

Pitcairn’s population avoided COVID-19-related hospitalizations and deaths despite its elderly population’s vulnerability to COVID-19, a significant level of comorbidities, and limited clinical management capabilities and options for emergency referrals. The pandemic highlighted some of the population’s health vulnerabilities while also underscoring some of their innate strengths.

## PROBLEM

On 30 January 2020, the World Health Organization (WHO) declared a public health emergency of international concern (PHEIC) following increasing transmission of severe acute respiratory syndrome coronavirus 2 (SARS-CoV-2), the pathogen that causes COVID-19. (*1*) The COVID-19 pandemic threatened all countries and areas, but the Pacific islands, including the remote Pitcairn Islands, had particular vulnerabilities in terms of baseline population health, access to care and health logistics. With only one doctor and one nurse to care for its ageing population of 30–40 residents and with limited referral options, Pitcairn faced unique challenges in preparing for and responding to the COVID-19 pandemic (Pitcairn Islands Census. 2022. Unpublished). Simultaneously, the territory’s extremely small size and strong community solidarity facilitated rapid decision-making and preparedness actions. This article describes Pitcairn’s unique context and how it effectively managed its COVID-19 pandemic response.

## CONTEXT

The Pitcairn Islands is an overseas territory of United Kingdom of Great Britain and Northern Ireland (UKOT) in the South Pacific. In 2023, the local resident population, exclusive of short-term government and contracted personnel, comprised 31 people, approximately half of whom were over 60 years old (Pitcairn Islands Census. 2022. Unpublished). As of May 2024, one child lives on the island. Variations in Pitcairn’s population are primarily due to deaths, schooling overseas, young adults working abroad, residents receiving long-term medical care overseas and occasional new settlers (**Table 1**).

**Table 1:** Pitcairn population census for 2020, 2021 and 2023

Age range	2020 (*n* = 34)	2021 (*n* = 34)	2023 (*n* = 31)
**0–17**	**6**	**4**	**0**
**18–30**	**3**	**3**	**5**
**31–40**	**1**	**1**	**0**
**41–50**	**6**	**4**	**3**
**51–60**	**4**	**7**	**6**
**61–70**	**11**	**10**	**10**
**71–80**	**1**	**2**	**4**
**81–90**	**1**	**2**	**2**
**91–100**	**1**	**1**	**1**
**Total no.**	**34**	**34**	**31**

*Source:* Pitcairn Island Censuses (unpublished).

**Fig. 1:**
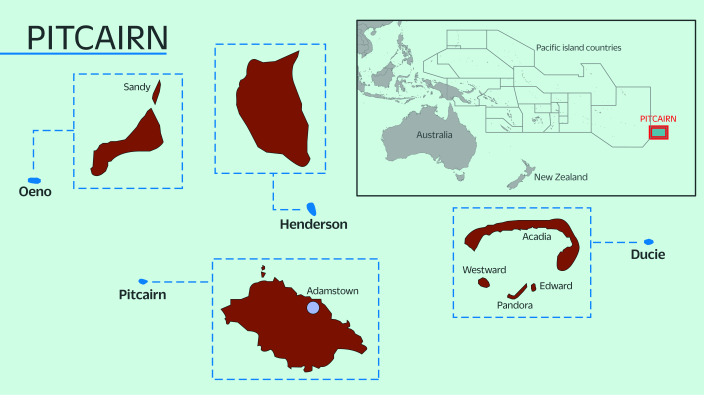
Map of the Pitcairn Islands

The Pitcairn Islands group comprises four islands – Pitcairn, Henderson, Ducie and Oeno – only one of which, Pitcairn, is inhabited. Made famous as a refuge for mutineers of the HMAV ***Bounty*** in 1790, the Pitcairn Islands group has a land mass of 47 km^2^. Only accessible by sea, the islands are located more than 2000 km from the nearest referral hospital in Tahiti, French Polynesia (**Fig. 1**). (*2*, *3*) Self-governance is enshrined in the territory’s Constitution, with the United Kingdom retaining responsibility for defence, foreign affairs and the provision of significant financial subsidies. Pitcairn’s local government is known as the Government of the Pitcairn Islands (GPI) and comprises an elected mayor and counsellors forming the Island Council (IC). (*4*)

[**insert** Figure 1]

The islands are some of the most remote in the world, with extremely challenging transportation logistics; for example, the nearest airport and seaport are 540 km away in Mangareva, French Polynesia, or approximately 36 hours by freighter ship. Pitcairn is currently served by a mixed passenger/cargo freighter chartered by the GPI. Capable of transporting up to 12 passengers per trip, it sails between Mangareva, French Polynesia or Tauranga, New Zealand and Pitcairn. (*5*) There are typically fewer than 20 passenger sailings per year.

Pitcairn has a health centre staffed by a local nurse employed by the Pitcairn IC and a medical officer (physician) contracted by the United Kingdom on rotation via the Pitcairn Island Office – an arrangement that has been in place since 2004. (*6*) The health centre is relatively well resourced in terms of medicines, medical equipment and supplies, which are procured from New Zealand or the United Kingdom. It operates primarily as an outpatient facility, with an examination room, radiographic imaging room, a resuscitation area, and a small area for overnight observation. Complex care, dental care and more advanced diagnostics are generally managed by referrals to French Polynesia or New Zealand.

While formal census data are not published, Pitcairn is probably the jurisdiction with the world’s smallest and oldest mean population (Pitcairn Island Health Centre. 2023. Unpublished). As of mid-2023, the island’s population included 31 adults, more than half of whom were over the age of 60, and nearly three quarters over the age of 50 (Pitcairn Island Census. 2022. Unpublished). With its rapidly ageing population, the health centre manages a high prevalence of chronic conditions, including asthma, chronic obstructive pulmonary disease, hypertension, diabetes, obesity and osteoarthritis. Nevertheless, given the island’s remote location and the need for self-sufficiency, the population’s lives are physically demanding. All residents engage in farming, transporting cargo to the island from the freighter ship, constructing and maintaining their homes and other structures, and helping maintain roads and other shared infrastructure.

The pandemic had a significant impact on Pitcairn’s economy and the livelihood of its population, which is heavily dependent on tourism, such as the sale of stamps and handicrafts to visitors, and the transport of passengers and freight aboard the United Kingdom’s MV *Silver Supporter*. (*7*)

The IC members were faced with the dual challenge of protecting the population’s health while maintaining its economic viability. Yet, Pitcairn’s small size and remoteness helped to delay the importation of SARS-CoV-2 and leveraged the time needed to achieve high vaccination coverage and to learn and adapt as the pandemic spread globally.

## ACTION

### Border and travel measures

Following WHO’s declaration of a PHEIC in January 2020 and the expansion of global SARS-CoV-2 transmission in February and early March, Pitcairn’s IC held a community meeting on 10 March, (*8*) and subsequently initiated strict border and travel measures on 12 March. (*9*)

From March 2020 to April 2022, Pitcairn’s borders were mostly closed, with very limited travel permitted for medical referrals and returning residents. Strict pre-departure quarantine, pre-departure polymerase chain reaction (PCR) testing, at-sea and on-arrival quarantine, and on-arrival rapid antigen testing were required. Pitcairn’s objective was to remain COVID-free for as long as possible, as there was very limited clinical management capacity on the island.

Pitcairn’s COVID-19 travel measures evolved progressively from the beginning of the pandemic and throughout the island’s multiple outbreaks. (*10*-*12*) These measures were based on the global epidemiological situation and the island’s risk assessments, which considered restrictions enforced by French Polynesia and New Zealand, from where the MV *Silver Supporter* would travel. As elsewhere in the world, Pitcairn’s leaders and community members discussed measures to balance the risks of COVID-19 importation, the arrival of vaccines and potential economic, social and other health impacts.

From early 2020 to mid-2022, overseas medical referrals were restricted, with only one referral to Tahiti in 2020. However, cargo shipping channels remained open.

### SARS-CoV-2 testing

In October 2020, Pitcairn received a donation of its first SARS-CoV-2 rapid antigen tests (RATs) from the French Polynesia’s Ministry of Health, facilitated by the WHO Regional Office for the Western Pacific’s Division of Pacific Technical Support and transported by Pitcairn Islanders returning from medical treatment in Tahiti. Subsequent supplies of RATs from the United Kingdom Foreign, Commonwealth and Development Office (FCDO) were delivered by the British Navy and later by freighter. Given the scale and staffing of its health centre and infection prevention and control requirements, Pitcairn residents were not able to access PCR testing on the island, although rapid antigen testing was initially carried out on all disembarking passengers. In late 2022 and early 2023, this was limited to those presenting with COVID-like symptoms. With support from WHO’s Division of Pacific Technical Support, PCR testing on Pitcairn was introduced in 2024.

### COVID-19 vaccine roll-out

The United Kingdom guaranteed delivery of COVID-19 vaccines to all UKOTs which, for Pitcairn, required a 15 000 km journey by air and sea. The IC anticipated the arrival of COVID-19 vaccines in January 2021, and arrangements were made for cold chain custody from ship to shore (Pitcairn Island Health Centre. 2021. Unpublished).

On 13 May 2021, a public meeting was held at Pitcairn’s Public Hall with the IC and 22 residents in attendance. Pitcairn’s Governor, Deputy Governor, and representatives of Public Health England (PHE) and the FCDO joined remotely via video link. The meeting informed the community on the incoming AstraZeneca vaccines, their safety and efficacy, vaccination requirements and other topics. (*11*)

COVID-19 vaccines arrived on Pitcairn on board the MV *Silver Supporter* on 17 May 2021. Initial uptake of the primary series of AstraZeneca vaccines was high at 37/44 residents. A second vaccine shipment arrived in February 2022 on board the Navy HMS *Spey* with Moderna vaccines, and 39/40 eligible persons were vaccinated, including three children. As of May 2023, nearly all of Pitcairn’s residents had completed a primary series and received two booster doses (Pitcairn Island Health Centre. 2023. Unpublished).

### Response support

Throughout the COVID-19 pandemic emergency period, Pitcairn’s medical officer and administrator on the island remained in contact with experts from PHE and the FCDO, and sought guidance from WHO regarding testing, quarantine and clinical management protocols. COVID-19 measures were regularly updated from 2020 to 2022. PHE experts facilitated by the FCDO provided technical advice and support to Pitcairn’s clinicians and the IC. Fortnightly or weekly telemedicine conferences were held with expertise shared between all UKOTs on SARS-CoV-2 testing, health policy, travel measures, quarantine, isolation, vaccination and response experiences, among others. Pitcairn’s physician and nurse also sought guidance from WHO and regularly reviewed measures and advice from government health authorities in Australia and New Zealand.

Beyond the provision of vaccines, SARS-CoV-2 testing supplies, personal protective equipment and technical guidance, the United Kingdom also provided Pitcairn’s residents with financial support through a COVID-19 debt support package to offset financial losses from the suspension of tourism. This fund, which also received a contribution from Pitcairn’s IC, provided permanent on-island residents with monthly credits of NZ$ 555.55, which could be used for purchases from the island’s general store, and for freight and general cargo shipping, loans and utilities. (*12*)

### Introduction of COVID-19 and community transmission

After achieving high vaccination coverage, Pitcairn’s borders were officially reopened on 31 March 2022 with regular travel resuming thereafter. After over 2 years of near-complete isolation, and gradual easing of border measures beginning in April 2022, residents faced their first cases of COVID-19 in July 2022 after two returning residents tested positive on arrival. Two close contacts were also infected. The passengers had tested negative before disembarking the MV *Silver Supporter* but tested positive later the same day on shore. All four patients were treated with antivirals (nirmatrelvir/ritonavir), and isolation protocols were established following consultation with the doctor, the IC and the administrator. No hospitalizations were required, and no deaths were recorded.

The Pitcairn IC adjusted measures to become COVID-safe rather than COVID-free, and regular passenger services resumed in July 2022 with 11 round-trip sailings between July 2022 and February 2023. Cruise ships resumed visits to Pitcairn in August 2022. (*13*) In March 2023, all remaining vaccination and testing requirements were lifted. While some residents queried the decision to reopen the border, consensus was eventually reached by weighing the risks and benefits to the population and the economy.

In April 2023, following visits from multiple cruise ships and yachts, a second COVID-19 outbreak was confirmed on the island. Following identification of an initial case, voluntary community rapid antigen testing was initiated, which reached nearly every resident. Approximately half of the island’s population tested positive, with 16 confirmed cases. Those presenting with symptoms, as well as older residents and those with comorbidities, were closely monitored by Pitcairn’s physician and nurse. (*14*)

As was the case in many countries and territories, Pitcairn adapted to the arrival of COVID-19. During outbreak periods, masks and hand sanitizer were provided to all residents, and mask wearing was encouraged in public areas. Outdoor IC meetings were held in the town square, and the general store temporarily adopted an electronic order and home-delivery service. Throughout both outbreaks, Pitcairn’s medical team communicated with all residents through the island’s radio system, which is connected to all homes. Patients did not present at the health centre but were asked to call for advice. When necessary, the nurse or doctor visited patients in their homes wearing personal protective equipment. RATs were carried out in patients’ homes or at an outdoor testing station next to the health centre.

In addition to radio communications, fact sheets based on New Zealand’s Public Health guidance were posted on notice boards in the town square, at the health centre and outside the general store. Supplies of masks and hand sanitizer were also available where people would congregate, such as in the general store.

## OUTCOMES

As of June 2024, Pitcairn was one of the only jurisdictions in the world not to have recorded a hospitalization or death related to COVID-19. This is attributed to: (i) the population’s small size and low probability of severe cases among fewer than 40 persons; (ii) the early and strict application of border and travel measures, which provided time for the vaccination of the population before cases were imported; and (iii) a high level of compliance with COVID-19 public health and social measures.

COVID-19 presented many medical, economic and social challenges for Pitcairn’s population. The long border closures had a significant impact on the island’s economy, though this was partially offset by support funds provided by the United Kingdom. Medical referrals, which are commonly required by the island’s ageing residents, became drastically more challenging. Many of the island’s residents were separated for a very long time from their families due to border measures in Pitcairn and abroad.

## Discussion

The Pitcairn IC, with support from the United Kingdom and partners, took decisive action in early 2020 to delay the importation of COVID-19 and continued those efforts during the vaccination of the population and the gradual easing of travel and other restrictions. Years of investment in Pitcairn’s health centre, the presence of a qualified physician and nurse on the island, and remote support from experts facilitated an effective response, which protected the health of the island’s population.

While Pitcairn avoided any COVID-19-related hospitalizations and deaths, the pandemic had significant secondary impacts, including delays in routine or non-urgent care for the population. For 2 years, Pitcairn’s population lacked access to offshore diagnostics and treatments, such as mammograms, joint replacements, chronic disease testing and cataract surgeries, among others.

While COVID-19 highlighted some of Pitcairn’s health vulnerabilities and underscored some of its innate strengths, the island’s ageing population and out-migration following the global easing of border and travel restrictions will continue to make population health risks more pressing. With fewer able-bodied residents and a physically challenging way of life, the continuity of the island’s core functions and ability to provide adequate and affordable health care to the population will require continued adaptation and innovation. (*15*)

Key lessons identified through Pitcairn’s COVID-19 preparedness and response efforts are listed below.

The maintenance of strong contact with the United Kingdom and other partners for technical guidance and support is important. Pitcairn’s remote location and isolation also underscore the importance of communications redundancy.
Pitcairn’s geographical isolation provided a degree of protection and permitted time for decision-making and the vaccination of most of the population before SARS-CoV-2 arriving on its shores.
Residents are well prepared for the possibility of a supply ship not arriving on schedule and are accustomed to being self-sufficient through fishing and farming. They had fewer concerns regarding the availability of essential goods than some populations who might have relied on imported goods.
Given the financial impact of the suspension of tourism for an extended period, the financial support of the United Kingdom was essential for the functioning of essential island operations and the well-being of the Pitcairn community.

### Limitations and future research

This article was developed through the collaborative effort of Pitcairn’s medical officer, on-island nurse and WHO’s Pitcairn Islands focal point, based on available data and information as well as reflections from first-hand experiences of coordinating preparedness and response efforts. No novel data collection was undertaken for this article. Opportunities remain for further research on the social and economic consequences of the COVID-19 pandemic on the island, as well as on the unique vulnerabilities and strengths of very small island communities in the face of public health threats.
